# Characterization of Spontaneous and TGF-β-Induced Cell Motility of Primary Human Normal and Neoplastic Mammary Cells In Vitro Using Novel Real-Time Technology

**DOI:** 10.1371/journal.pone.0056591

**Published:** 2013-02-14

**Authors:** Katharina Mandel, Daniel Seidl, Dirk Rades, Hendrik Lehnert, Frank Gieseler, Ralf Hass, Hendrik Ungefroren

**Affiliations:** 1 Biochemistry and Tumor Biology Laboratory, Gynecology Research Unit, Department of Gynecology and Obstetrics, Medical University, Hannover, Germany; 2 First Department of Medicine, University Hospital Schleswig-Holstein (UKSH), Campus Lübeck, Lübeck, Germany; 3 Department of Radiation Oncology, University Hospital Schleswig-Holstein (UKSH), Campus Lübeck, Lübeck, Germany; Wayne State University School of Medicine, United States of America

## Abstract

The clinical complications derived from metastatic disease are responsible for the majority of all breast cancer related deaths. Since cell migration and invasion are a prerequisite for metastasis their assessment in patient cancer cells in vitro may have prognostic value for the tumor's metastatic capacity. We employed real-time cell analysis (RTCA) on the xCELLigence DP system to determine in vitro motility of patient-derived primary human breast cancer epithelial cells (HBCEC). Initially, the RTCA assay was validated using established human breast cancer cell lines with either an invasive (MDA-MB-231, MDA-MB-435s) or a non-invasive phenotype (MCF-7, MDA-MB-468), and primary NSCLC cells (Tu459). Previous standard assays of cell migration/invasion revealed that only MDA-MB-231, −435s, and Tu459 cells exhibited spontaneous and TGF-β1-stimulated migration and invasion through a Matrigel barrier. In the present study, the TGF-β1-stimulated activities could be blocked by SB431542, a potent kinase inhibitor of the TGF-β type I receptor ALK5. Application of the RTCA assay to patient-derived tumor cells showed that 4/4 primary HBCEC and primary NSCLC cells, but not normal human mammary epithelial cells (HMEC), displayed high spontaneous migratory and invasive activity which correlated with higher MMP-2 expression and uPA protein levels in HBCEC compared to HMEC. Upon treatment with TGF-β1, HBCEC exhibited morphologic and gene regulatory alterations indicative of epithelial-to-mesenchymal transition. However, exclusively the invasive but not the migratory activity of HBCEC was further enhanced by TGF-β1. This indicates the requirement for molecular, e.g. integrin interactions with Matrigel components in HBCEC in order to become responsive to pro-invasive TGF-β effects. Together, these results show for the first time that tumorigenic HBCEC but not normal HMEC possess a strong basal migratory as well as a basal and TGF-β1-inducible invasive potential. These findings qualify the RTCA assay as an in vitro migration/invasion testing system for patient-specific primary breast cancer cells.

## Introduction

Breast cancer is the most common cancer in women and a major cause of morbidity and mortality. Worldwide, approximately 350,000 women die from breast cancer each year [1]. A challenging problem is the high mortality due to the spread of tumor cells to distant organs, particularly, liver, lungs, bones or the brain [2]. The development of metastatic disease is responsible for the majority of deaths. In order to metastasize, cancer cells must progress through a series of steps, which together are termed the metastasis cascade [3]. Cell invasion represents an initial step in this cascade and the ability of epithelial cells at the tumor margins to migrate away from the primary site is an early determinant of the transition from an in situ towards an invasive phenotype. Since metastasis cannot occur without initial migration/invasion, the invasive capacity of cells represents a major determinant of their metastatic potential. Hence, a better understanding of the migratory mechanisms used by cells is important for our understanding of some key events influencing mortality in breast cancer [4].

Tumor cell spreading and metastasis depend on the local hypoxic microenvironment and on the interaction with adjacent neighboring cells including mesenchymal stem cells, tumor-associated macrophages and cancer-associated fibroblasts [5]–[13]. This process is also largely controlled by environmental non-genetic factors (soluble and solid) present in the tumor microenvironment including cytokines, chemokines and growth factors. In breast cancer, transforming growth factor (TGF)-β has been shown to play an essential role in generating a metastatic phenotype by stimulating an epithelial-mesenchymal transition (EMT), cell migration, invasion and bone and lung metastasis, and in modifying the microenvironment to the advantage of cancer cells [14]. Within the highly regulated process of invasion the mesenchymal cancer cells are remodeling the ECM of the invaded tissue by expressing and secreting high amounts of matrix-degrading enzymes such as urokinase-type plasminogen activator (uPA) and matrix metalloproteinases (MMPs). The plasminogen activator system is composed of important proteolytic enzymes not only for fibrinolysis but also for extracellular matrix remodeling. The protease uPA and its natural inhibitor, plasminogen activator inhibitor-1 (PAI-1), have been implicated in breast cancer metastasis whereby these two enzymes contribute to the degradation of extracellular matrix components liberating certain tumor cells for enhanced migration and distal invasion. Therefore, uPA and PAI-1 serve as separate prognostic factors in clinical tests for patients with node-negative and medium-grade breast cancer [15]. With regard to MMPs, the relative expression level of MMP-2 in tissues of invasive ductal breast carcinomas was significantly higher than that of adjacent non-tumor tissues [16]. Blocking MMP-2 secretion and activation during breast carcinoma development may decrease metastasis [17]. Moreover, MMP-2 upregulation is induced by TGF-β and associated with TGF-β-induced EMT [18] and invasion [19] in breast epithelial cells. During progression tumor cells may experience various alterations in TGF-β signaling that enhance the ability of this growth factor to stimulate cell invasion and metastasis [14].

Breast cancer patients are currently treated with standardized chemotherapy, endocrine treatment and/or radiation therapy which can all have serious side effects as a result of perturbation of proliferation-active tissue homeostasis. The process of metastasis is the prime target for molecular therapeutics, however, the response to antimetastatic agents depends on the histological type and pattern of molecular alterations of each tumor, but even if this knowledge is available, it is hard to predict whether the tumor cells are invasive and will eventually become metastatic. One way to resolve this problem is to bring cells obtained from biopsies in primary culture and to characterize their migratory and invasive properties in vitro. This approach might allow for an estimation of their metastatic capacity in vivo and thus may prove to be a potent prognostic tool for their malignant potential. Recently, Hass and Bertram have shown that primary human breast cancer epithelial cells (HBCEC) derived from explant cultures of patient tumor biopsies maintain tumor cell characteristics in long term in vitro culture [20], [21] and also in vivo using immune-deficient NOD/SCID mice. However, whether HBCEC are capable of migration and invasion in vitro is not known currently and remains to be tested.

Traditionally, cell migration and spreading has been measured using transwell or scratch assays, however, both assay types provide only limited kinetic information as they represent endpoint assays and are not easily quantifiable. The novel real-time cell analysis (RTCA) assay (applied on the xCELLigence DP system) is based on changes in electrical impedance at the electrode/cell interphase [22] and compensates for these drawbacks by allowing to monitor cell migration in real-time and without the incorporation of labels. In a recent study, the xCELLigence RTCA technology has been systematically compared with transwell/Boyden chamber assays of cell migration and invasion using MDA-MB-231 breast cancer cells and was found to exhibit strong correlations [23]. This general validation allows for a retrospective comparison of RTCA data with data from conventional assays for measurements of cell motility and largely removes the need for internal validation. In contrast to chemotaxis which measures directional cell migration induced by a chemoattractant, chemokinesis characterizes the spontaneous and non-directed migratory and invasive properties. Recently, we have successfully applied the RTCA chemokinesis assay to the study of migration-associated signaling events in pancreatic carcinoma cells [24]–[26].

We have therefore applied the RTCA assay in a migration (chemokinesis) and invasion setup to primary human normal and neoplastic mammary cells under both steady-state and TGF-β1-stimulated conditions. Prior to application of this assay to HBCEC we have taken care to validate it against established breast cancer cell lines (MDA-MB-231, MDA-MB-435s) and primary non small cell lung cancer (NSCLC) cells with a known migratory and invasive phenotype. We show here that 4/4 primary HBCEC, but not normal human mammary epithelial cells (HMEC), displayed high migratory and invasive activity. Notably, and in contrast to the MDA-MB-231 and −435 s cell lines only the invasive activity of HBCEC was further enhanced by exogenous TGF-β1. This is the first study that provides real-time data on spontaneous and growth factor-mediated cell migration and invasion kinetics of primary human breast cancer cells.

## Materials and Methods

### Ethics Statement

Individual HBCEC were isolated from small tissue biopsies of breast cancer patients diagnosed for invasive ductal carcinoma (HBCEC #335 and HBCEC #338), lobular breast carcinoma (HBCEC #360) and the rare case of a phyllodes tumor (HBCEC #699). The study has been approved by the institutional ethics committee of the Medical University of Hannover, Germany, Project #3916 on June 15th, 2005. Tu459 cells were derived from the pleural effusion of a lung cancer patient diagnosed for NSCLC. The study has been approved by the institutional ethics committee of the Medical Faculty of the University of Kiel, Germany, Project Az:D421/04 on June 30th, 2004 and informed written consent was obtained from the patient.

### Mammary Epithelial Cell Cultures

HMEC were commercially obtained from Lonza Walkersville, Inc. (Walkersville, MD, USA). Primary epithelial cells from a 50 year old caucasian female were isolated from mammary tissue. Cells were received as culture passage 6 (P6) (Lot #92796) and were tested negative for HIV-1, yeast, fungi, bacteria and hepatitis B & C. HMEC were cultured under humidified conditions at 37°C, 5% CO_2_ in serum-free mammary epithelial basal medium (MEBM) (Lonza, Cologne, Germany). The medium was supplemented with 52 µg/ml of bovine pituitary extract, 0.5 µg/ml of hydrocortisone, 5 µg/ml of human recombinant insulin and 10 ng/ml of human recombinant epidermal growth factor and was substituted every three to four days. At subconfluency, HMEC were passaged as described previously [27]. Briefly, cells were washed with HepesBSS buffer (Lonza) and incubated with 0.025%/0.01% trypsin/EDTA (Lonza) for 6 min at 37°C. Following complete detachment of cells (which was monitored microscopically), trypsin inactivation with trypsin neutralization solution (TNS) (Lonza) and centrifugation (500 g for 6 min), approximately 4,500 cells/cm^2^ were transferred into a new culture flask. In the experiments HMEC of passage 11 and 12 were used.

### Isolation and Culture of Primary Human Breast Cancer-derived Epithelial Cells (HBCEC) and Tu459 NSCLC Cells

Individual HBCEC were isolated from small tissue biopsies of breast cancer patients. Explant cultures of the tumor biopsies were established in uncoated plastic dishes (Nunc GmbH, Langenselbold, Germany) in a humidified atmosphere (37°C, 5% CO_2_) and the outgrowth of primary HBCEC was subcultured after about 3–4 weeks as described previously [20]. The primary tumor-derived cells used in this study were HBCEC #335 (invasive ductal carcinoma, grading 1, (pT1c, pN1, Her2−); HBCEC #338 (invasive ductal carcinoma, grading 3, (pT1c, pN0, Her2 3+)); HBCEC #360 (lobular carcinoma, grading 2, (pT1c, pN1, Her2−)); and HBCEC #699 (55-year-old patient with benign phyllodes breast tumor). Histopathologic inspection of the primary HBCEC #699 explants revealed mixed epithelial-fibroblastoid cell structures consistent with the description of phyllodes tumors as fibroepithelial tumors of the breast [28]. HBCEC underwent the same testing procedure for contamination and were cultured under the same conditions as described above for HMEC except that they were maintained initially at a density of 1,500 cells/cm^2^. All HBCEC were cultured in subconfluent conditions similar to HMEC and were used for the experiments in passage 2 or 3.

Tu459 cells were isolated in our laboratory from the pleural effusion of a 62-year old male patient with metastasized NSCLC (squamous cell carcinoma, stage IV) [29]. The cells were cultured in DMEM supplemented with 10% fetal bovine serum (FBS), 2 mM L-glutamine and penicillin/streptomycin and were used in passage 12–13.

### Culture of Established Breast Cancer Cell Lines

The established human breast carcinoma cell lines MCF-7 (HTB-22), MDA-MB-231 (HTB-26), MDA-MB-435s (HTB-129), and MDA-MB-468 (HTB-132), were originally purchased from ATCC and routinely maintained in RPMI 1640 (Lonza) supplemented with 10% FBS, 2 mM L-glutamine, 2 mM sodium pyruvate and penicillin/streptomycin. Prior to the experiments the three MDA-MB cell lines underwent a short tandem repeat (STR)-based authentication by the Institute for Legal Medicine at the University Hospital Schleswig-Holstein. The results of the STR matching analysis (http://www.dsmz.de/fp/cgi-bin/str.html) confirmed the identity of the respective cell lines. All cells used in this study were routinely tested for the absence of mycoplasma contamination using the MycoAlert Mycoplasma Detection Kit from Lonza.

### Migration and Invasion Assays

Real-time cell analysis (RTCA) of migration and invasion was performed on the xCELLigence DP device (Roche Diagnostics, Mannheim, Germany) essentially as described in the supplier's instruction manual and in Ref. 21. Briefly, cells were added to the upper chamber of a two-chamber device separated by a porous membrane (the CIM-plate 16) and either attach and migrate directly through the pores to the bottom side of the membrane (where the electrodes reside) or do so following initial invasion through a solid matrix. In either setup this leads to an increase in electrical impedance of integrated gold microelectrodes. The electrical impedance is displayed as a dimensionless parameter termed cell index. The cell index represents the capacity for cell migration or invasion, and the slope of the curve can be related to the migration or invasion velocity of tumor cells. The cell index thus reflects the tumor cell's migratory and invasive capacity, e.g., invasion by highly aggressive cells leads to large changes in cell impedance and vice versa. Prior to cell seeding the bottom side of the wells from the upper chamber of the CIM-plate 16 was coated with 30 µl of collagen I (400 µg/ml, Sigma, Deisenhofen, Germany). 30,000–50,000 cells/well suspended in culture medium containing 1% FBS were then seeded in the upper chamber according to the manufacturer's manual (Roche Diagnostics). The invasion assay was performed similarly, except that the surface of the upper chamber was covered with a monolayer of 5% (v/v) growth factor-reduced Matrigel (BD Biosciences, Heidelberg, Germany, diluted 1∶20 with basal medium as detailed by Roche in the application notes). In some experiments, 10 ng/ml TGF-β1 (stock solution: 10 µg/ml in 10 mM citric assay, pH 3.0, 0.1% bovine serum albumin, RELIATech, Wolfenbüttel, Germany) and 5 µM of the activin receptor-like kinase 5 (ALK5) inhibitor SB431542 (stock solution: 10 mM in DMSO, Calbiochem/Merck; Darmstadt, Germany) or vehicle-only (0.05% DMSO) [30] were added to both the lower and upper wells at the same concentration. Cell indices were measured every 15 min for up to 48 h with the RTCA software (version 1.2, Roche Diagnostics).

### RNA Isolation and Quantitative Real-time RT-PCR (qPCR) Analysis

Total RNA was isolated from breast cancer cell lines using PeqGoldRNApure (peqlab, Erlangen, Germany) and was reverse-transcribed with Superscript II reverse transcriptase (Invitrogen). The conditions for qPCR as well as PCR oligonucleotide primer sequences for MMP-2, E-cadherin, N-cadherin, vimentin, biglycan, Snail, Slug and the housekeeping genes TATA-box binding protein (TBP) and β-actin have been described elsewhere [31], [32]. The qPCR was performed on an I-cycler with IQ software (Bio-Rad, München, Germany). All values for the genes of interest were normalized to that of TBP and β-actin, and relative gene expression was calculated by the 2^−ΔΔ^Ct method.

### ELISA for Human uPA and Human PAI-1

A quantitative determination of uPA and PAI-1 in protein homogenates of the different breast cell populations was performed by appropriate enzyme-linked immunosorbent assays (ELISAs) as described previously [33], [34]. Briefly, cell cultures of the primary HMEC and HBCEC populations as well as the breast cancer cell lines MDA-MB-231 and MCF-7 were frozen in liquid nitrogen and pulverized in the frozen state using a microdismembrator (Braun). The resulting powder was suspended in extraction buffer (American Diagnostica GmbH, Pfungstadt, Germany) and the determination of protein content was performed by the colorimetric BCA-assay (Perbio Science Deutschland, Bonn, Germany). The protein homogenates of the different breast culture samples were appropriately diluted and the subsequent ELISA assay was performed by a protocol according to the manufacturer's instructions (American Diagnostica GmbH). The detection limit was 25 pg/ml for the uPA ELISA and 125 pg/ml for the PAI-1 ELISA.

### Statistical Analysis

Statistical significance was calculated using the unpaired Student's *t*-test. Data were considered significant at *p*<0.05.

## Results

### Real-time Measurement of Cell Migration and Invasion in Various Established Breast Cancer Cell Lines

**Figure 1:**
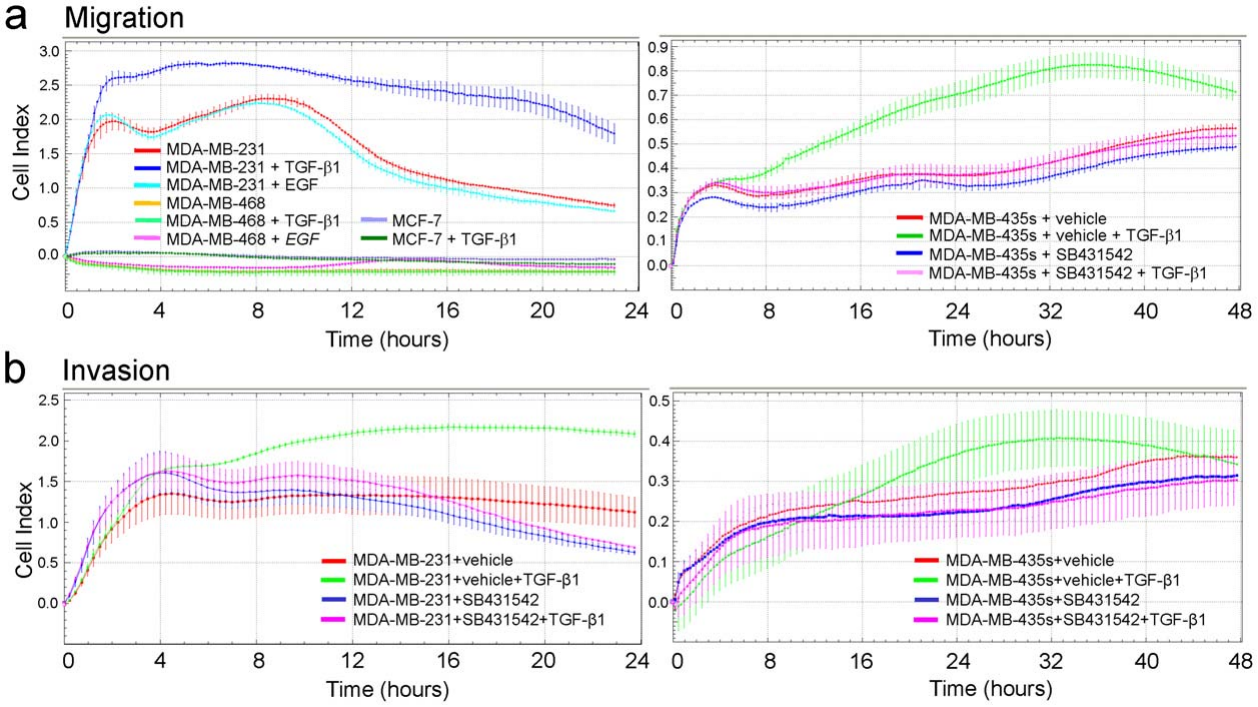
Real-time measurement of cell migration and cell invasion in various established breast carcinoma cell lines using the xCELLigence DP system. **a**, left panel, Migration analysis of MDA-MB-231, MDA-MB-468, and MCF-7 cells. Cells were stimulated with either TGF-β1 (5 ng/ml) or EGF (20 ng/ml) as indicated by the color code in serum-reduced medium during the assay. At the 8, 16, 24, 32, and 40 h time points differences were highly significant (p<0.001) between control (red curve) and TGF-β1-treated (blue curve) but not between control and EGF-treated (cyan curve) MDA-MB-231 cells. Right panel, Migration curves of MDA-MB-435s cells in the presence of the ALK5 inhibitor SB431542 (5 µM) or vehicle-only (0.05% DMSO). At the 16, 24, 32, and 40 h time points differences were highly significant (p<0.001) between SB431542+TGF-β1-treated (pink curve) and vehicle+TGF-β1-treated (green curve) MDA-MB-435s cells. **b**, Matrigel invasion RTCA assay with MDA-MB-231 cells (left panel) and MDA-MB-435s cells (right panel) with or without TGF-β1 stimulation (5 ng/ml). Both cell lines were treated with TGF-β1 in the presence of either vehicle-only (0.05% DMSO) or the ALK5 inhibitor SB431542 (5 µM). In order to prepare the CIM-Plates 16 for the invasion setup, the bottom of the upper compartment of each well was covered with a thin layer of Matrigel prior to cell seeding. Data are depicted as mean ± SD of three or four wells processed in parallel. In each panel, a representative experiment is shown. At the 8, 12, 16, and 20 h time points differences were highly significant (p<0.001) between control (red curve) and TGF-β1-treated (green curve) MDA-MB-231 cells. Differences were significant (p<0.05) between SB431542+TGF-β1-treated cells (pink curve) and vehicle+TGF-β1-treated cells (green curve) at the 12, 16, and 20 h time points (MDA-MB-231, left panel) and at the 24, 32, and 40 h time points (MDA-MB-435s, right panel).

To perform an internal validation of the xCELLigence platform for the measurement of cell motility in breast cancer cell lines, RTCA cell migration and invasion assays were performed with established breast cancer cell lines. MDA-MB-231 and MDA-MB-435s cells are both highly metastatic and have been shown to exhibit cell migration in vitro as measured with conventional assays, while others are non-metastatic and possess a non-migratory phenotype (MDA-MB-468 and MCF-7). As expected from results with transwell assays from other studies, MDA-MB-231 (Fig. 1a, left graph) and MDA-MB-435s (Fig. 1a, right graph) cells displayed high basal migratory activity (as indicated by the increases in cell-index values, essentially reflecting the amount of migration-active cells), while the other cell lines failed to migrate under these conditions (MCF-7, MDA-MB-468: Fig. 1a, left graph). Regarding the kinetics, MDA-MB-231 cells migrated rapidly within the first 4–6 h (as reflected by the steep slope of the curves), and reached a plateau (MDA-MB-231: Fig. 1a, left graph) In contrast, MDA-MB-435s presented with slower migration kinetics, peaking at approx. 36 h (Fig. 1a, right graph), and cell-index values were lower than those of MDA-MB-231 cells. In order to show that the RTCA assay is also a suitable tool to monitor changes in the migratory kinetics that result from the stimulation of cells with migration promoting or inhibiting agents, we treated cells with TGF-β1. In previous publications we have shown that this factor strongly increased the migratory and invasive behavior of pancreatic ductal adenocarcinoma cells in RTCA assays [24]–[26]. Interestingly, MDA-MB-231 (Fig. 1a, left graph) and −435s cells (Fig. 1a, right graph) responded to TGF-β1 stimulation with strongly enhanced migration while MDA-MB-468 and MCF-7 cells were refractory in this respect (Fig. 1a, left graph). The pro-migratory effect in MDA-MB-231 cells was specific to TGF-β1 since treatment of cells with Epidermal growth factor (EGF), a known inducer of breast cancer cell chemotaxis [35] did not enhance migration (Fig. 1a, left graph). The migratory response to exogenous TGF-β1 was sensitive to the ALK4/5/7 inhibitor SB431542 which completely abolished TGF-β1-induced cell motility in both MDA-MB-231 (data not shown) and MDA-MB-435s cells (Fig. 1a, right graph), indicating that the TGF-β1 effect was mediated by the TGF-β type I receptor ALK5.

Next, we performed a series of invasion assays with MDA-MB-231, MDA-MB-435s, and MCF-7 cells and Matrigel as a barrier. Pilot experiments using different Matrigel dilutions (1∶40, 1∶20, 1∶10) indicated a density-dependent decrease in cell-index (data not shown). All further experiments were performed with a 1∶20 dilution of the Matrigel. Both MDA-MB-231 (Fig. 1b, left graph) and MDA-MB-435s cells (Fig. 1b, right graph) but not MCF-7 cells (data not shown) were able to invade the Matrigel albeit with different kinetics (MDA-MB-231: fast, MDA-MB-435s: slow). As observed for migration, the ability of both cell types to invade the Matrigel was further enhanced by TGF-β1 and the TGF-β1 effect was completely inhibited by SB431542 (Fig. 1b).

Together, these results show that the migration and invasion behavior in vitro of MCF-7 and MDA-MB-468 cells on the one hand, and MDA-MB-231 and MDA-MB-435s cells on the other hand correlates well with their low and high, respectively, metastatic capacity in vivo. Together, these data show that the RTCA assays can reproduce the results from conventional migration and invasion assays with breast cancer cells with respect not only to basal but also to growth factor receptor stimulated and corresponding kinase inhibitor suppressed cell motility.

### Real-time Measurement of Cell Migration in HBCEC and HMEC

**Figure 2:**
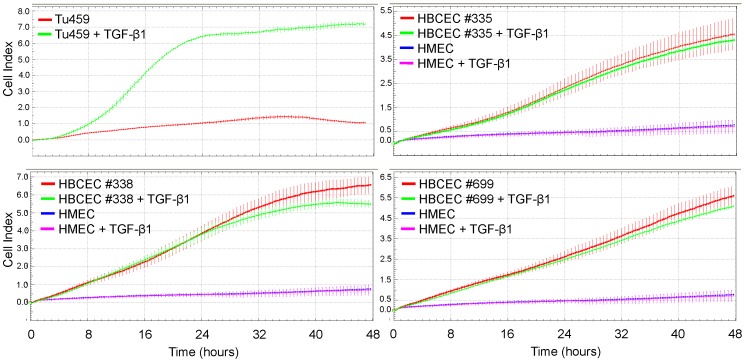
RTCA migration assay with Tu459 NSCLC cells, HBCEC, and HMEC. A total of 50,000 cells of each population were seeded per well in the upper chamber of a CIM-Plate 16. Cells were left untreated or were stimulated with TGF-β1 (10 ng/ml). The three HBCEC were compared to the same HMEC. Data shown represent the mean ± SD of three-four wells processed in parallel. For Tu459 cells, HBCEC #335 and #699 a representative experiment from 3 experiments is shown, for HBCEC #338 an experiment with data intermediate between those of two other experiments run with identical conditions is presented. Differences were highly significant (p<0.001) at the 8, 16, 24, 32, and 40 h time points between untreated HBCEC (red curves) and untreated HMEC (blue curves).

Based upon the results obtained with the established breast cancer cell lines it was of interest to demonstrate the utility of the xCELLigence platform for assessing the migratory activity of primary human normal and tumorigenic mammary cells. For this purpose we first employed primary Tu459 NSCLC cells which exhibit strong migratory activity in conventional transwell assays (H. U., unpublished observation). As expected, these cells also presented with basal and strong TGF-β1-induced migratory activity in the RTCA assay (Fig. 2, upper left panel) and the latter was efficiently blunted by treatment of the cells with SB431542 (data not shown).

Following extensive validation of this technology for the measurement of cell motility in established breast cancer cell lines and primary lung cancer cells, we performed another series of migration and invasion chemokinesis assays with HBCEC and HMEC using the same conditions as described above. Four different HBCEC (#335, #338, #360, #699) were analysed in the migration assay under both steady-state and TGF-β1-stimulated conditions. In vivo experiments after subcutaneous injection of these primary cells into NOD/SCID mice revealed no detectable tumor formation with normal young proliferating HMEC (P12) and the phyllodes breast tumor-derived HBCEC #699. In contrast, injection of invasive ductal breast carcinoma-derived HBCEC led to a significant tumor growth within about 6 weeks (data not shown).

Interestingly, the HBCEC populations #335, #338, and #699 (Fig. 2) as well as #360 (data not shown) exhibited strong migratory activities when measured over a period of 48 h. Conversely, HMEC (P12) which have been characterized previously as young and proliferation-active cells [27] were used as a control in this assay since these normal breast epithelial cells did not generate a detectable migration signal (Fig. 2). Notably, all four HBCEC were unresponsive to TGF-β1 treatment during the first 24 h (HBCEC #335, #338, #699: Fig. 2, HBCEC #360: not shown), although the response of HBCEC #338 was somewhat variable in this respect (data not shown). Again, HMEC remained migration-negative even after exposure to TGF-β1 (Fig. 2). The migratory activity of HBCEC was independently confirmed and verified directly by time-lapse video microscopy (data not shown). Together, the results depicted in Fig. 2 show that HBCEC, but not HMEC, exhibited strong migration potential in vitro and that TGF-β1 was unable to further promote cell migration in HBCEC and HMEC in contrast to Tu459 NSCLC cells.

### Real-time Measurement of Cell Invasion

**Figure 3:**
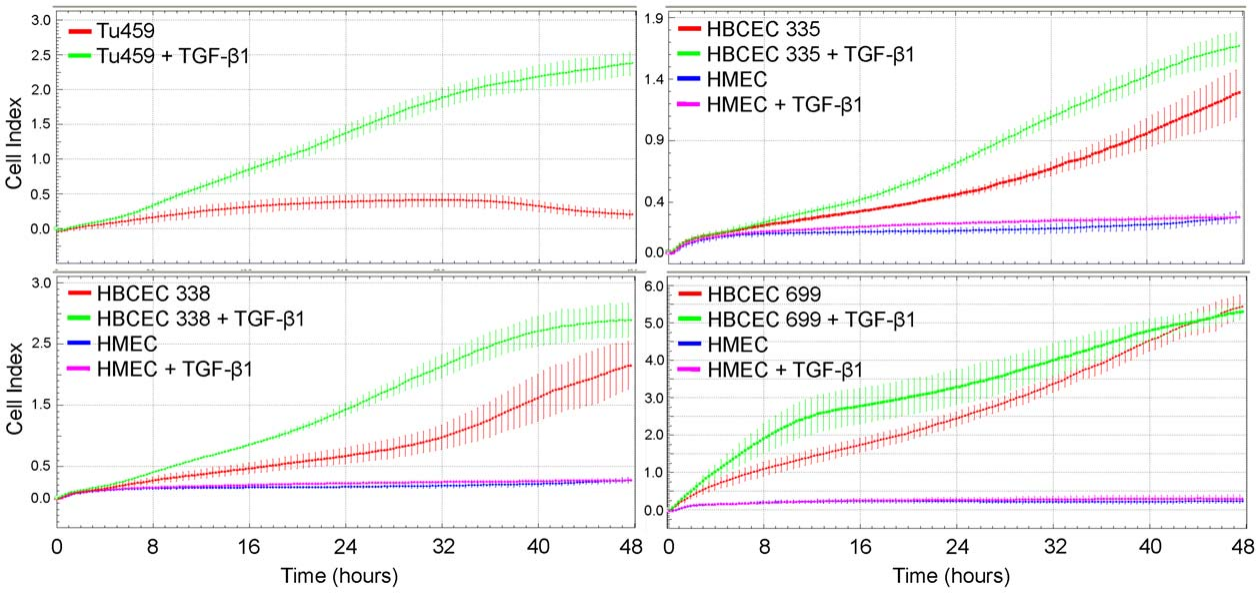
RTCA invasion assay with Tu459 NSCLC cells, HBCEC, and HMEC. A RTCA assay was performed with 50,000 cells/well from each cell population in CIM-Plates 16 precoated with Matrigel. Cells were left untreated or were stimulated with TGF-β1 (10 ng/ml). In each graph HBCEC were compared to the same HMEC. Data shown represent the mean ± SD of three or four wells processed in parallel. A representative experiment from 3 experiments performed is shown. Differences between control and TGF-β1-treated cells were significant at the 24, 32, and 40 h time points for HBCEC #335, the 16, 24, 32, and 40 h time points for HBCEC #338, and the 8, 16, 24, and 32 h time points for HBCEC #699.

The capacity for cell invasion is a prerequisite for tumor cells to abandon the primary tumor and to colonize distant sites in the human body. The xCELLigence technology also allows for testing the invasive capacity of tumor cells when covering the microporous membrane separating the upper from the lower chamber with a solid matrix as barrier, e.g. Matrigel. Validation of the RTCA invasion assay with primary Tu459 NSCLC cells demonstrated that these cells presented with both basal and TGF-β1-induced invasion (Fig. 3, upper left panel). Next, we tested the HBCEC #335, #338, and #699 in the invasion setup of the RTCA assay. All three primary HBCEC were capable of invading through Matrigel with the following order of cell indices: #699>#338>#335 (Fig. 3). Notably, TGF-β1 strongly enhanced invasion of all three HBCEC (Fig. 3). As expected from the migration data, HMEC did not show any signs of invasive behavior neither under steady-state conditions nor after exposure to exogenous TGF-β1 (Fig. 3). These data indicate that HBCEC exhibit a strong invasion potential and that TGF-β1 was clearly effective in stimulating Matrigel invasion of HBCEC but not HMEC.

### Determination of EMT in HBCEC and HMEC

**Figure 4:**
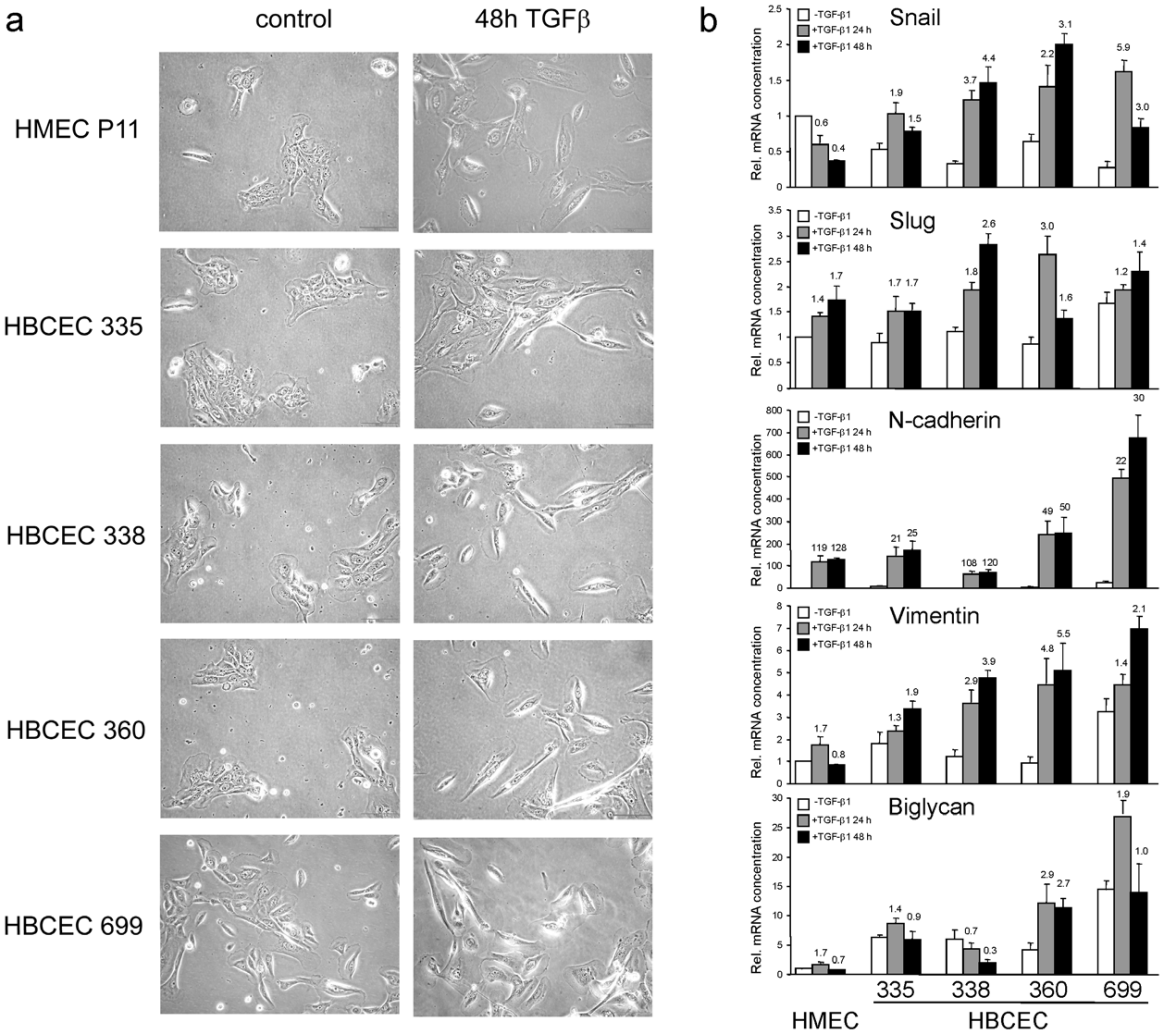
Determination of EMT in HBCEC and HMEC. **a**, Microscopic analysis of HMEC and various HBCEC after stimulation with TGF-β1 (10 ng/ml) for 48 h. P3 = passage 3. Bar, 100 µM. **b**, Expression of the indicated EMT-associated marker genes in the same HMEC and HBCEC shown in **a**. Data represent the mean ± SD of three reactions after normalization with the housekeeping genes TBP and β-actin.

**Figure 5:**
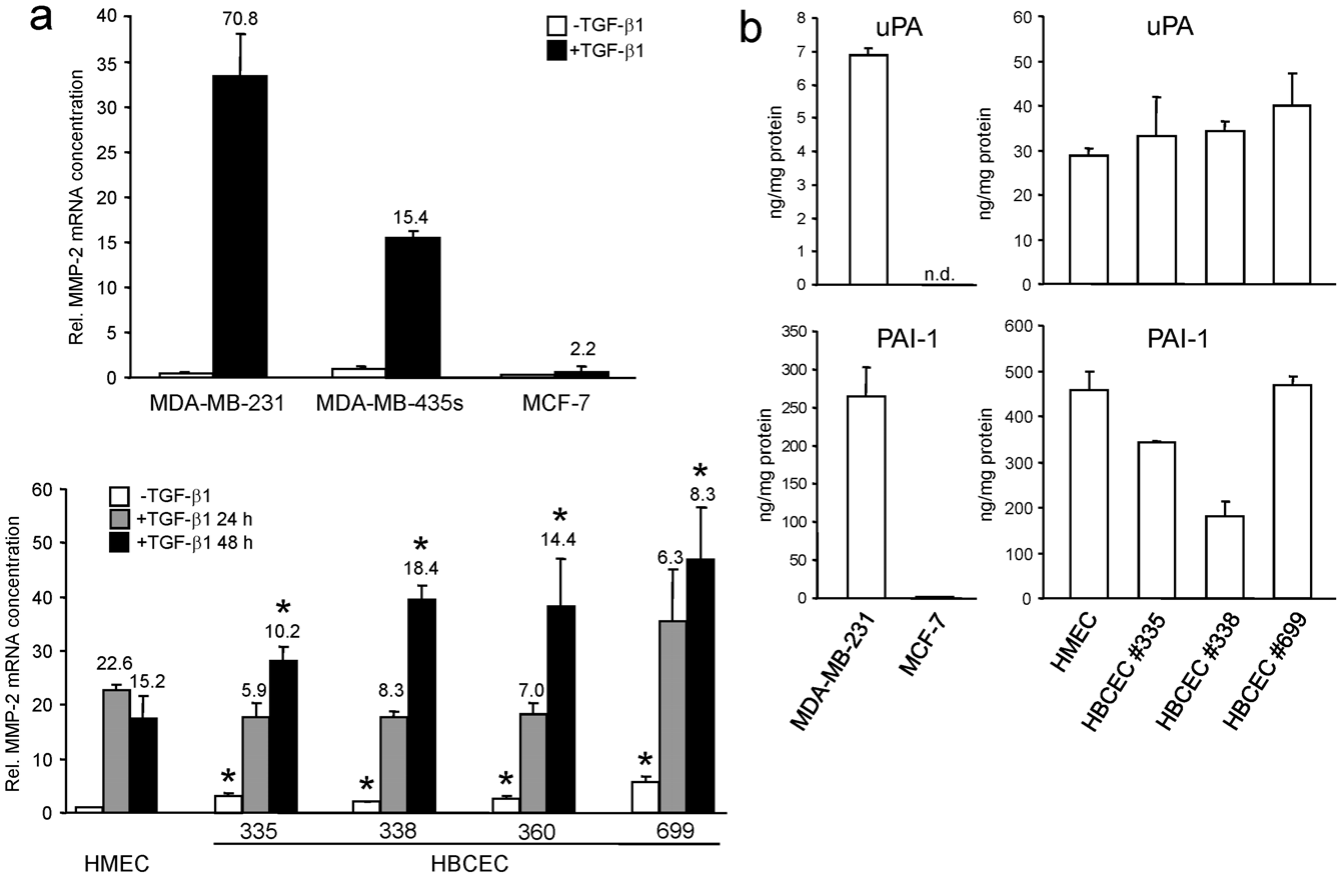
Expression of MMP-2, uPA and PAI-1 in HBCEC, HMEC, and permanent breast cancer cell lines. **a**, QPCR determination of MMP-2 expression in MDA-MB-231, MDA-MB-435s, and MCF-7 cells (upper graph) and in HMEC and various HBCEC (lower graph) after stimulation with TGF-β1 (10 ng/ml) for 24 h and 48 h. Data are presented as mean ± SD of three reactions after normalization with the housekeeping genes TBP and β-actin. The numbers above the graphs indicate -fold induction by TGF-β1 relative to unstimulated controls. Asterisks indicate significant differences relative to the respective treatment groups in HMEC. **b**, Expression of uPA (upper graphs) and PAI-1 (lower graphs) in MDA-MB-231 and MCF-7 cells (left graphs), and HMEC and HBCEC (right graphs) as measured by ELISA. Data represent the mean ± SD of duplicate determinations. (n = 2 to 3). n.d., not detectable.

EMT is thought to be a prerequisite for tumor cells to become motile and eventually invasive and metastatic. Both the MDA-MB-231 and −435 s cell lines have been shown to undergo EMT in response to TGF-β1 stimulation [36]. To test whether this is also the case for primary HBCEC and HMEC, we assessed the cell morphology and the expression of various EMT-associated marker genes after 24 h and 48 h of treatment with TGF-β1 (Fig. 4). Unstimulated control cells of HBCEC #335, #338, and #360 grew as small clusters of tightly packed flat and rounded cells with typical epithelial-like cell morphology (Fig. 4a). In contrast, cultures of HBCEC #699 contained a larger number of elongated cells and cell clusters appeared more loosened (Fig. 4a). After 48 h of TGF-β1 treatment, however, all four HBCEC and the HMEC cultures grew as single lying cells rather than cell clusters and many cells adopted a spindle-shaped morphology (Fig. 4a). In HBCEC but not HMEC TGF-β1 upregulated the expression of EMT-associated transcription factors including Snail, while the related Slug and the mesenchymal markers N-cadherin, biglycan, vimentin (Fig. 4b), and MMP-2 (Fig. 5a) were induced by TGF-β1 in both HMEC and HBCEC. The steady-state and TGF-β1-induced expression of these genes was generally higher and more persistent in HBCEC as compared to HMEC. Expression of the epithelial marker E-cadherin was similar in HMEC and HBCEC and was not altered by TGF-β1 at the transcriptional level (data not shown). Among the different primary tumor cultures, the phyllodes tumor-derived HBCEC #699 behaved differently since these cells exhibited the highest steady-state expression for all markers tested except Snail (Figs. 4b and 5a). Together, these data suggest that both HBCEC and HMEC undergo EMT in response to TGF-β1.

### Determination of MMP-2 Expression and uPA and PAI-1 Levels in Established Breast Cancer Cell Lines and in HBCEC and HMEC

The marked effects of TGF-β1 on cell invasive activities of HBCEC (see Fig. 3) suggest that TGF-β1 contributes to the activation of extracellular matrix proteases such as MMP-2 thus facilitating cell invasion through Matrigel. As evidenced by qPCR, the expression level of MMP-2 after TGF-β1 stimulation was significantly more elevated in highly invasive MDA-MB-231 and MD-MB-435s cells as compared to non-invasive MCF-7 cells (Fig. 5a). To test whether a similiar scenario could be observed in migration/invasion-active primary breast cancer cells *versus* non-motile normal breast epithelial cells, we measured MMP-2 expression in the various HBCEC and in HMEC. In contrast to HMEC, all four HBCEC displayed an increased MMP-2 expression at steady-state conditions and after 48 h of TGF-β1 treatment (Fig. 5a).

As already mentioned in the Introduction, uPA and PAI-1 serve as separate prognostic factors for certain breast cancer patients with respect to a potential therapeutic assessment [15]. Interestingly, uPA and PAI-1 levels appear to be different between patient-derived primary breast cancer cells and immortalized breast cancer cell lines (Fig. 5b). Thus, MDA-MB-231 cells demonstrated detectable amounts of uPA and PAI-1 whereas levels of these proteins were near the detection limit in MCF-7 cells (Fig. 5b, left graphs). In contrast, the amount of uPA was markedly higher in HBCEC #338 and #699 compared to HMEC (Fig. 5b, top right graph). Conversely, the amount of the corresponding inhibitor PAI-1 was significantly lower in the tumorigenic HBCEC #335 and #338 as compared to HMEC, and the latter showed a similar level to that observed for HBCEC #699 (Fig. 5b, bottom right graph). Together these data suggest that both higher MMP-2 expression and higher uPA levels in HBCEC as compared to HMEC may contribute to their enhanced constitutive invasion potential. Our findings further suggest that the HBCEC RTCA assay could represent an in vitro migration and invasion testing system for patient-specific breast tumor-derived primary cells and that their behavior may be exploited as a prognostic factor for tumor aggressiveness and potentially metastatic capacity.

## Discussion

Here we present, for the first time, data on the migratory and invasive activities of primary cultures from breast cancer patients using the principle of electric cell-substrate impedance sensing. The RTCA assay carried out with the xCELLigence DP system represents a novel real-time-based cell migration assay which allows for continuous data recording over a period of several days. In contrast to endpoint assays, the RTCA assay allows for the precise determination of the kinetics of migratory and invasive activity of a given cell population in culture.

Since no previous data were available on how breast cancer cells behave in the RTCA assay (except for MDA-MB-231 cells [23]), we initially validated this technology with breast cancer cell lines of a known metastatic phenotype. We employed established mammary carcinoma lines including some with low (MCF-7, MDA-MB-468) and high (MDA-MB-231, MDA-MB-435s) metastatic potential and observed that the migratory and invasive potential in vitro corresponded well with their metastatic behavior in vivo in that only MDA-MB-231 and MDA-MB-435s cells were positive in this assay. In addition, the RTCA revealed differences in migration kinetics between MDA-MB-231 and −435s cells that would have hardly been detected in endpoint assays.

Since in vitro and in vivo studies have shown that TGF-β promotes breast carcinogenesis by inducing EMT, invasion, and metastasis [4], [14], [19], [36], we analysed whether the permanent breast cancer cell lines (and primary Tu459 NSCLC cells as control) were capable of responding to TGF-β1 with increased migration and invasion in the RTCA assay. Only MDA-MB-231 cells, MDA-MB-435s cells and Tu459 cells responded with an increase in both migration and invasion. As previously shown for pancreatic ductal adenocarcinoma cell lines, the pro-migratory effect of TGF-β1 could be inhibited with SB431542, indicating that it was mediated by the TGF-β type I receptor ALK5. SB431542 also slightly reduced basal motility in both breast cancer cell lines (Fig. 1b), suggesting participation of autocrine TGF-β and/or activins in driving cell motility. The invasion-promoting effect of TGF-β1 observed in MDA-MB-231 and MDA-MB-435s, but not in MDA-MB-468 and MCF-7 cells, is consistent with the finding that MMP-2 expression was much more strongly induced by TGF-β1 in MDA-MB-231 and MDA-MB-435s compared to MCF-7 cells (see Fig. 5a) and with the lack of uPA and PAI-1 expression in MCF-7 cells (see Fig. 5b). Together, these data clearly show that the xCELLigence technology can reproduce the results from conventional migration and invasion assays with respect not only to basal activity but also to growth factor receptor stimulation and corresponding kinase inhibitor-mediated suppression of cell motility in breast and lung cancer cells.

We then applied these RTCA assay conditions to the analysis of migration and invasion of four different primary breast tumor populations (HBCEC) and observed that they exhibited both spontaneous migratory and invasive activities. In contrast, HMEC failed to exhibit any migratory or invasive activity under these conditions which may partially been explained by a lower expression of MMP-2, and a lower level of uPA along with a higher level of PAI-1 (see below) compared to HBCEC. Interestingly, the two patients from which HBCEC #335 and #360 were generated, presented with lymph node metastases (N1) whereas the patient of HBCEC #338 albeit being classified N0 was at high-risk in developing metastatic disease (grading 3, pT1c, pN0, Her2 3+). Also of note, despite being diagnosed as benign tumor and the lack of detectable tumor formation in NOD/SCID mice within 3 months, HBCECs #699 exhibited strong motility in the RTCA assays. This can possibly be explained by the mixed epithelial/fibroblastoid morphology of the cells from primary explant cultures (see Fig. 4a). Moreover, these cells exhibit a comparatively high constitutive and TGF-β1-inducible expression of some mesenchymal markers (see Figs. 4b and 5a) which is also substantiated by the high uPA levels (see Fig. 5b).

The uPA and PAI-1 assay in the clinical practice is used at the highest level of evidence for node-negative mammary carcinoma with intermediate grading, whereby uPA (cut-off at 3 ng/mg protein; Femtelle) and PAI-1 (cut-off at 14 ng/mg protein; Femtelle) levels are evaluated separately. Whereas uPA represents one of the extracellular matrix proteases contributing to matrix digestion and thus cellular motility, this protease activity can be reduced by high levels of its corresponding inhibitor PAI-1 as observed in HMEC. However, one should be cautious to directly compare levels of these proteins between primary cells and established cell lines from the same tumor entity. For MDA-MB-231 cells, the uPA and PAI-1 levels may be different due to artificial immortalization and establishment as a degenerated cell line. Therefore, aberrant expression levels of certain genes in MDA-MB-231 cells which have been described previously may also provide the activation of alternative/additional extracellular matrix protease systems besides uPA/PAI-1 to enable migration and invasion of these cells. In this context, previous work has demonstrated that a variety of MMPs are constitutively expressed in MDA-MB-231 but not in normal breast epithelial cells [37].

Surprisingly, exogenous TGF-β1 failed to enhance migration in the HBCEC populations when the chemokinesis/migration setup of the RTCA assay was applied. There was even a tendency towards an *inhibitory* effect at later times (>24 h), although it should be mentioned that here the migratory activity may have already been obscured by TGF-β1-induced growth inhibition. Intriguingly, however, 3/3 HBCEC reproducibly responded to TGF-β1 with an increase in cell invasion through Matrigel. At times >24 h the curve slopes of TGF-β1-treated cells intersected with those of control cells possibly indicating growth inhibition as discussed for the respective migration data. The reason for the inability of exogenously applied TGF-β1 to enhance migration and invasion in HMEC and migration in the absence of a solid matrix in HBCEC remains unclear. It does not reflect a general unresponsiveness of these cells to TGF-β, since the same cells responded to TGF-β1 with EMT, increased MMP-2 expression, and even enhanced invasion in case of HBCEC. However, TGF-β-induced cell motility is known to require crosstalk with integrin signaling in order to drive appropriate changes in gene expression ([38] and references therein). It is therefore likely that the Matrigel but not the CIM-plate 16 microporous membrane itself or its collagen coating provides integrin ligands that are crucial for TGF-β-driven cell motility in primary breast cancer cells, a hypothesis that is currently being tested in our laboratory.

Taken together, the results of this study show for the first time that primary human breast cancer cells, in contrast to non-tumorigenic HMEC, display high basal spontaneous migratory and invasive activities, the latter of which can be further enhanced by TGF-β1 stimulation. HBCEC are suitable for the characterization of individual cancer cells and in vitro testing of anti-migratory/anti-metastatic agents required for a personalized anti-tumor therapy. Since proper diagnosis is mandatory for individually adjusting cancer therapeutics to late-stage breast cancer patients, results from RTCA assays may aid in correctly diagnosing metastatic tumors based on patient samples. The xCELLigence DP system thus provides a useful tool to assess the degree of malignancy in patient-specific breast cancer cells.
