# Correction: Characterization of Spontaneous and TGF-β-Induced Cell Motility of Primary Human Normal and Neoplastic Mammary Cells In Vitro Using Novel Real-Time Technology

**DOI:** 10.1371/annotation/e799099f-9509-4769-b761-853fccec4541

**Published:** 2013-04-24

**Authors:** Katharina Mandel, Daniel Seidl, Dirk Rades, Hendrik Lehnert, Frank Gieseler, Ralf Hass, Hendrik Ungefroren

There was an error in Funding statement. The correct statement is:

This work was sponsored in part by the Niedersächsische Krebsgesellschaft (awarded to Ralf Hass). The funders had no role in study design, data collection and analysis, decision to publish, or preparation of the manuscript. 

